# Involvement of HisF in the Persistence of *Acinetobacter baumannii* During a Pneumonia Infection

**DOI:** 10.3389/fcimb.2019.00310

**Published:** 2019-08-29

**Authors:** Marta Martínez-Guitián, Juan C. Vázquez-Ucha, Laura Álvarez-Fraga, Kelly Conde-Pérez, Cristina Lasarte-Monterrubio, Juan Andrés Vallejo, Germán Bou, Margarita Poza, Alejandro Beceiro

**Affiliations:** Servicio de Microbiología Do Complexo Hospitalario Universitario da Coruña (CHUAC), Instituto de Investigación Biomédica da Coruña (INIBIC), Universidade da Coruña (UDC), A Coruña, Spain

**Keywords:** HisF, mice pneumonia model, lung infection, *Acinetobacter baumannii*, virulence

## Abstract

*Acinetobacter baumannii* is currently considered one of the most problematic nosocomial microorganisms. In the present work the *hisF* gene from the ATCC 17978 strain and the AbH12O-A2 clinical isolate of *A. baumannii* was found over-expressed during the course of murine pneumonia infections. The study demonstrated that the *A. baumannii* ATCC 17978 mutant strain lacking the *hisF* gene induces a sub-lethal pneumonia infection in mice, while the complemented mutant strain increased its virulence. This histidine auxotroph mutant showed an increase on IL-6 secretion and leukocytes recruitment during infections. Furthermore, data revealed that the *hisF* gene, implicated in the innate immunity and inflammation, is involved in virulence during a pneumonia infection, which may partly explain the ability of this strain to persist in the lung. We suggest that HisF, essential for full virulence in this pathogen, should be considered a potential target for developing new antimicrobial therapies against *A. baumannii*.

**Importance**

Nosocomial pathogens such as *A. baumannii* are able to acquire and develop multi-drug resistance and represent an important clinical and economic problem. There is therefore an urgent need to find new therapeutic targets to fight against *A. baumannii*. In the present work, the potential of HisF from *A. baumannii* as a therapeutic target has been addressed since this protein is involved in the innate inmunity and the inflamatory response and seems essential to develop a pneumonia in mice. This work lays the groundwork for designing antimicrobial therapies that block the activity of HisF.

## Introduction

*Acinetobacter baumannii* is included in a list published by the WHO of the most important antibiotic-resistant bacteria (World Health Organization, 2017). This species shows a high capacity to persist in hospital environments and to develop antimicrobial resistance. There is therefore an urgent need to find new therapeutic targets for designing novel strategies for fighting against this pathogen.

The *hisF* gene of *A. baumannii* is involved in histidine and *de novo* purine biosynthesis. The *hisH* and *hisF* products shape the heterodimeric protein imidazole glycerol phosphate (ImGP) synthase. This heterodimeric enzyme catalyzes transformation of the intermediate N′-(5′-phosphoribosyl)-formimino-5-aminoimidazol-4-carboxamide ribonucleotide (PRFAR) into 5′-(5-aminoimidazole-4-carboxamide) ribonucleotide (AICAR) and ImGP, which are further used in *de novo* purine and histidine biosynthesis, respectively (Figure S1).

One of the products of HisF, AICAR, an analog of adenosine monophosphate (AMP), is capable of stimulating AMP-activated protein kinase (AMPK) activity. Both AICAR monophosphate and AMP, which are small molecules, trigger a conformational change in the AMPK complex that allows further activation by phosphorylation of Thr-172 (Kim et al., 2016). The AMPK enzyme, the central regulator of energy homeostasis, participates in the cellular response to metabolic stress and is considered an important therapeutic target for controlling different human diseases (Kim et al., 2016). Once activated, AMPK phosphorylates numerous metabolic enzymes, causing global inhibition of biosynthetic pathways and activation of catabolic pathways and thus generating and conserving energy (Ruderman et al., 2003). As well as stimulating AMPK, AICAR can also inhibit the lipopolysaccharide-induced production of proinflammatory cytokines (Giri et al., 2004). Treatment with an adenosine kinase inhibitor was able to block the ability of AICAR to activate AMPK, preventing inhibition of inflammation in mice mesangial cells (Jhun et al., 2004; Peairs et al., 2009). Other authors have also described the role of AICAR in regulating inflammation (Giri et al., 2004; Jhun et al., 2004; Peairs et al., 2009).

In the present study, we found that the *hisF* gene was over-expressed in the lungs of mice with pneumonia caused by the ATCC 17978 strain and the multiresistant AbH12O-A2 clinical isolate of *A. baumannii*. The aim of the study was to investigate the involvement of the *hisF* gene in the pathogenesis of *A. baumannii* by using *in vitro* and *in vivo* assays.

## Materials and Methods

### Microbial Strains and Culture Conditions

*A. baumannii* ATCC 17978 and its mutant derivative strains, as well as the AbH12O-A2 clinical isolate of *A. baumannii* and *E. coli* TG1 (Table 1) were routinely grown or maintained in Luria-Bertani (LB) or Mueller-Hinton (MH) media supplemented with 2% agar when necessary. All strains were grown at 37°C and stored at −80°C in LB broth with 10% glycerol. When appropriate, cultures were supplemented with kanamycin (km) at a final concentration of 50 mg/L (Sigma-Aldrich, USA).

**Table 1:** Bacterial strains and plasmids used in this study.

**Strain or plasmid**	**Relevant characteristics**	**Source or references**
**STRAIN**		
***A. baumannii***		
ATCC 17978	Clinical isolate	ATCC^*^
Δ*hisF*	A1S_3245 gene (*hisF*) deletion mutant obtained from the ATCC 17978 strain	This study
ATCC 17978 + pWH1266-Km	ATCC 17978 harboring the empty pWH1266-Km plasmid; Km^R^, Tet^R^	This study
Δ*hisF* + pWH1266-Km	Δ*hisF* harboring the empty pWH1266-Km plasmid; Km^R^, Tet^R^	This study
Δ*hisF* complemented	Δ*hisF* harboring the pWH1266-Km-*hisF* plasmid; Km^R^	This study
AbH12O-A2	Multiresistant clinical isolate, which caused a large nosocomial outbreak	Merino et al., 2014; Pérez et al., 2017
***E. coli***		
TG1	Used for DNA recombinant methods	Lucigen
**PLASMID**		
pWH1266-Km	*A. baumannii* shuttle vector; Km^R^, Tet^R^	Álvarez-Fraga et al., 2016
pWH1266-Km-*hisF*	pWH1266-Km harboring the A1S_*hisF* gene; Km^R^	This study
pMo130	Suicide vector for the construction of *A. baumannii* isogenic derivative; Km^R^, SacB, XylE	Hamad et al., 2009

* *American Type Culture Collection*.

### Determination of the *hisF* Gene Expression

An experimental model of pneumonia in mice was used to describe the transcriptome of the ATCC 17978 and the multiresistant AbH12O-A2 clinical strains of *A. baumannii* during the course of infection, as previously reported (Álvarez-Fraga et al., 2018). Briefly, BALB/c male mice were intratracheally inoculated with ~6 × 10^7^ bacteria *per* mouse. Bronchoalveolar lavage (BAL) was performed at 20 h to obtain bacteria for RNA extraction (*in vivo* samples). RNA extracted from bacteria grown in LB medium was used as an experimental control (*in vitro* samples). Total RNA was used for RNAseq analysis (Illumina, Biogune, Spain). Reads from each mRNA library were obtained using HiScanSQ (Illumina Inc., CIC bioGUNE, Bilbao, Spain). Gene expression analysis was done at CIC bioGUNE's genome analysis platform (Derio, Spain). Raw data were deposited in the GEO database with the accession code GSE100552 (document named GSE100552_ATCC_ODvsATCC _raton.tsv.gz).

### Construction of the Isogenic Mutant Strain

The *hisF* gene was identified as A1S_3245 in the first genome sequence of *A. baumannii* ATCC 17978 (CP000521) (Fernando et al., 2014) and later annotated as AU097_05240 in a recent genome sequence of this strain (CP018664.1). The isogenic mutant strain (Δ*hisF*), lacking the *hisF* gene, was derived from the *A. baumannii* ATCC 17978 strain by double crossover recombination using the suicide vector pMo130 (Genbank: EU862243), as previously described (Álvarez-Fraga et al., 2016). Briefly, the Δ*hisF* isogenic mutant derivative was constructed by deleting a fragment of the A1S_3245 gene. The upstream and downstream regions flanking the A1S_3245 gene were PCR-amplified and cloned into the vector pMo130 using the primers shown in Table S1. The construction obtained was used to transform wild type cells by electroporation. Recombinant colonies were selected as previously described (Álvarez-Fraga et al., 2016). The second crossover event was checked by PCR using primers listed in Table S1.

### Complementation of the Mutant Strain

In order to obtain the complemented strain, the A1S_3245 gene was PCR-amplified from the genome of the ATCC 17978 strain and cloned into vector pWH1266-Km using the primers shown in Table S1. The pWH1266-Km plasmid was constructed as previously described (Hamad et al., 2009; Álvarez-Fraga et al., 2016). The genetic construction obtained was used to transform the Δ*hisF* isogenic mutant strain. The complemented Δ*hisF* mutant strain was selected in kanamycin-containing plates. The ATCC 17978 and the Δ*hisF* strains harboring the empty pWH1266-Km vector were used as experimental controls.

### Biofilm Formation Assay

Biofilm formation ability was assessed as described by Tomaras et al. (2003) and modified by Álvarez-Fraga et al. (2016). Briefly, cells of *A. baumannii* centrifuged from an overnight culture were washed and resuspended in LB medium. Then, samples were stagnant incubated at 37°C for 48 h. Growth was monitored at OD_600nm_ and the amount of biofilm formed was quantified by crystal violet staining using ethanol-acetone. The OD_580nm_/OD_600nm_ ratio was used to normalize the amount of biofilm formed to the total cell content of each sample.

### Adhesion to A549 Human Alveolar Epithelial Cells

The ability of the strains to adhere to A549 human epithelial cells was evaluated as described by Gaddy et al. (2009) and modified by Álvarez-Fraga et al. (2016). Briefly, A549 human cells were grown at 37°C and 5% CO_2_ in Dulbecco modified Eagle medium (DMEM) (Sigma-Aldrich) containing 1% of penicillin-streptomycin (Fisher Scientific, UK) and 10% of fetal bovine serum. Monolayers were washed with HBSS (Hank's balanced salt solution, Fisher Scientific) without glucose (mHBSS). A549 cells (1 × 10^5^ cells/well) were then infected with 10^7^ bacteria in 24-well plates and incubated for 3 h in mHBSS at 37°C. Finally, A549 cells were washed and lysed with sodium deoxycholate. Lysates were plated and incubated at 37°C for 24 h. Colony forming units (CFUs) were counted to determine the number of bacteria adhered to cells.

### Growth Curve Analysis

Fitness was assessed by measuring the growth rates of the ATCC 17978 strain, the isogenic mutant strain Δ*hisF*, the ATCC 17978 + pWH1266-Km, the Δ*hisF* + pWH1266-Km, and the Δ*hisF* mutant strain complemented as described before. Briefly, 1.5 ml of LB medium was inoculated with ~5 × 10^7^ CFU of each strain, previously grown until the stationary phase, and incubated at 37°C with shaking. Growth was monitored in an Epoch 2 Microplate Spectrophotometer (BioTek Instruments, Inc.) and OD_600nm_ values were recorded every 20 min as previously described (Álvarez-Fraga et al., 2018).

Growth curves were also performed to demonstrate the histidine auxotrophy of the Δ*hisF* mutant. To perform these assays, 10 × 10^5^ CFUs/well of the ATCC 17978 strain and the isogenic mutant strain Δ*hisF* were grown in M9 minimal medium in presence or absence of 0.5 mM of histidine. Cultures were inoculated as above described. Samples were collected at 3, 6, 24, and 48 h and the number of viable bacteria present in the medium was checked by colonies counting in LB agar plates.

### Determination of A549 Cells Survival Infected With *A. baumannii* Strains

An experimental model of infection of A549 human alveolar epithelial cells was used to study the *in vitro* virulence of *A. baumannii* strains as previously described (González-Bello et al., 2015). Briefly, cells were cultured in DMEM suplemented with 10% fetal bovine serum and 1% of penicillin-strptomycin. Later, 1 × 10^5^ cells per well were infected with 5 × 10^7^ CFUs of parental and Δ*hisF* mutant strain and incubated 20 h at 37°C. The number of inoculated bacteria was determined by direct plating. A LIVE/DEAD fluorescence microscopy kit (Cellstain Double-staining Kit; Fluka, Switzerland) was used according to the manufacturer's instructions to measure cell viability post-infection. The A549 cells were incubated for 15 min at 37°C with the two fluorescent molecules to obtain simultaneous fluorescent staining; calcein-AM, to stain viable cells (green), and propidium iodide, to stain only dead cells (red). Microscopic images of the stained cells were obtained using an inverted fluorescence microscope (Nikon Eclipse Ti) and analyzed with the NIS Elements Br software package.

### Susceptibility Testing

Antimicrobial susceptibility analyses, done by the disk diffusion method, were performed using ampicillin, cefoxitin, ceftazidime, cefepime, imipenem, tigecycline, rifampicin, colistin, ciprofloxacin, and gentamicin disks (Sigma-Aldrich) (Clinical and Laboratory Standards Institute, 2018).

### Murine Pneumonia Model

A murine pneumonia model was used to examine the role of the *hisF* gene in *in vivo* virulence. Briefly, BALB/c mice were intratracheally inoculated with 40 μL of a bacterial suspension containing 3 × 10^9^ CFU/mL sterile saline solution and 10% of porcine mucin (wt/vol) (Sigma-Aldrich) mixed at 1:1 ratio (Álvarez-Fraga et al., 2018).

To ascertain the relevance of the *hisF* gene in virulence using a murine pneumonia model, a second assay was performed to determine the bacterial burden in lungs. Groups of 8 mice were intratracheally inoculated, as above described, with ATCC 17978 wild type and Δ*hisF* mutant strains. Mice were sacrificed at 20 h and lung samples were obtained and processed as previously described (Rodríguez-Hernández et al., 2000).

### Murine Sepsis Model

A murine sepsis model was also constructed using BALB/c mice. Mice were inoculated intraperitoneally with 100 μL of a bacterial suspension containing 75 × 10^7^ CFU/mL, as previously described (Beceiro et al., 2014). The survival rate was assessed during 7 days post-infection.

### *Galleria mellonella* Infection Model

The virulence of the ATCC 17978 wild type and Δ*hisF* mutant strains was assessed using a *Galleria mellonella* infection model as previously described (Álvarez-Fraga et al., 2018). Briefly, *G. mellonella* caterpillars (Bio Systems Technology, UK) were infected with 2 × 10^4^ bacteria and incubated at 37°C in the dark. Death was determined every 8 h during 6 days to obtain the larval survival rates.

### IL-6 Production Determination

Cell-free supernatants from infected RAW 264.7 cells and from BAL fluid from infected mice were used to analyse cytokine IL-6. RAW 264.7 macrophages were grown at 37°C and 5% CO_2_ in DMEM medium (Sigma-Aldrich) containing 10% fetal bovine serum and 1% penicillin-streptomycin (Fisher Scientific). An amount of 1 × 10^5^ RAW 264.7 cells *per* well were infected with 3.5 × 10^7^ CFU of the parental or mutant strains using 24-well plates. Cell supernatants were collected at 2, 6, and 20 h post-infection, centrifuged at 1,000x *g* for 10 min and stored at −80°C prior to IL-6 production determination.

BAL fluids from lungs of mice infected as above described with the wild type or the mutant strain were extracted at 6 and 24 h after the challenge. A protease inhibitor cocktail was added and immediately centrifuged at 1,000x *g* for 10 min.

IL-6 was measured in both macrophages and lung samples by ELISA using the Murine IL-6 ELISA Kit (Diaclone, France) as previously described (Alnahas et al., 2017).

### Leukocyte Counts

In the murine pneumonia model, BAL fluid was obtained 6 and 24 h after the challenge, to determine the total leukocyte cell counts. Cells were fixed and stained with Diff-Quick Stain (Thermo-Scientific, USA). Counts were performed using a microscope (Olympus, Japan) and the software cellSens Dimension (Olympus).

### Phagocytic Assays

The phagocytic activity was assessed against the *A. baumannii* strains used in this study. RAW 264.7 murine cells were maintained in DMEM supplemented with 10% fetal bovine serum and 1% of penicillin-streptomycin. Macrophages were activated RAW 264.7 during 3 days in presence of phorbol 12-myristate 13-acetate (PMA, Sigma-Aldrich) at 100 nM. Then macrophages RAW 264.7 were scraped and seeded into 48-well plates (4 × 10^4^ cells/well). Bacterial strains were cultured in LB or LB with 50 mg/L of kanamycin at 37°C until 0.7 OD_600nm_, washed twice in saline solution and adjusted at 10 × 10^6^ CFU/mL in DMEM + 10% FBS. Additionally, AICAR at 1 mM was added to the Δ*hisF* mutant and incubated 30 min at room temperature. Infections were performed adding 200 μL of bacterial inoculum to each well with cells and incubated 1 h at 37°C. Finally, supernatants were aspirated and plated onto LB or LB plus kanamycin plates. The determination of phagocytosis activity was calculated by comparison with control wells with bacterial inoculum without the presence of macrophages.

### Statistical Analysis

A student's *t*-test was used to evaluate the statistical significance of the observed differences in all assays, except in the survival assays, in which the survival curves were plotted by the Kaplan-Meier method and analyzed with the log-rank test. Differences were considered statistically significant at *p* ≤ 0.05. All assays were performed at least in triplicate.

### Ethics Statement

All experiments with mice were performed in accordance with regulatory guidelines and standards established by the Animal Ethics Committee (CHUAC, Spain, project code P82).

## Results and Discussion

Expression analysis of the *hisF* gene was performed using the *A. baumannii* ATCC 17978 strain, one of the best-studied strains of this species, and the multiresistant AbH12O-A2 clinical isolate, that caused more than 300 colonizations/infections in a hospital in Madrid, Spain (Merino et al., 2014; Pérez et al., 2017). Transcriptomic analysis using RNAseq Illumina procedures revealed that, relative to bacteria grown *in vitro*, the *hisF* gene was over-expressed in both the ATCC 17978 (7.2-fold) and the AbH12O-A2 clinical (19.2-fold) *A. baumannii* strains, causing lung infection in mice.

Expression of other genes involved in histidine biosynthesis was also checked. The pathway of histidine biosynthesis is complex and involves nine genes (*hisG, hisI, hisE, hisA, hisF, hisH, hisB, hisC*, and *hisD*) (Fani et al., 1998). Among these genes, only *hisF* was found to be over-expressed during the lung infection, in both the ATCC 17978 and the AbH12O-A2 clinical strains. A key step occurs when the formation of two products is catalyzed by the heterodimeric enzyme complex ImGP synthase, which consists of *hisH* and *hisF*. One of the products, ImGP, is further used in histidine biosynthesis, and the other, AICAR, is used in *de novo* synthesis of purines and AMPK activation (Fani et al., 1998; O'Donoghue et al., 2001) (Figure S1). Thus, AICAR synthesis mainly depends on HisH and HisF. The mechanism whereby HisH produces ammonia (NH_3_) from glutamine involves hydrolysis to release NH_3_ and glutamate (Chittur et al., 2001). The sequential HisH and HisF reactions are strongly coupled in order to facilitate the necessary transfer of NH_3_ to the HisF active site and to produce ImGP and AICAR. In our *in vivo* transcriptomic analysis, we detected over-expression of the *hisF* gene whereas the expression level of the *hisH* gene remained unaltered. The different speed of reaction of these enzymes could explain the relevance of HisF in AICAR production and in virulence during the lung infection. Thus, it has been demonstrated that a 5,300-fold increase in basal glutamine hydrolysis produced by HisH is observed in the presence of the substrate PRFAR (Myers et al., 2003). In this case, over-expression of *hisH* may not be as important as over-expression of *hisF* for increasing the AICAR synthesis.

An isogenic mutant strain lacking *hisF* gene (Δ*hisF*) derived from the ATCC 17978 strain was constructed in order to investigate the function of *hisF*. No significant differences between the ATCC 17978 strain and its derivate isogenic mutant Δ*hisF* were observed in biofilm formation or capacity for adherence to A549 human alveolar epithelial cells (Figures S2A,B). There was also no difference in growth rate between the parental and the Δ*hisF* mutant strain (Figure S2C) or in the survival rate of human alveolar epithelial A549 cells infected with the mutant and the wild type strains (Figure S2D). No changes in susceptibility to any of the tested antimicrobials were detected when the *hisF* gene was deleted (Table S2).

**Figure 1:**
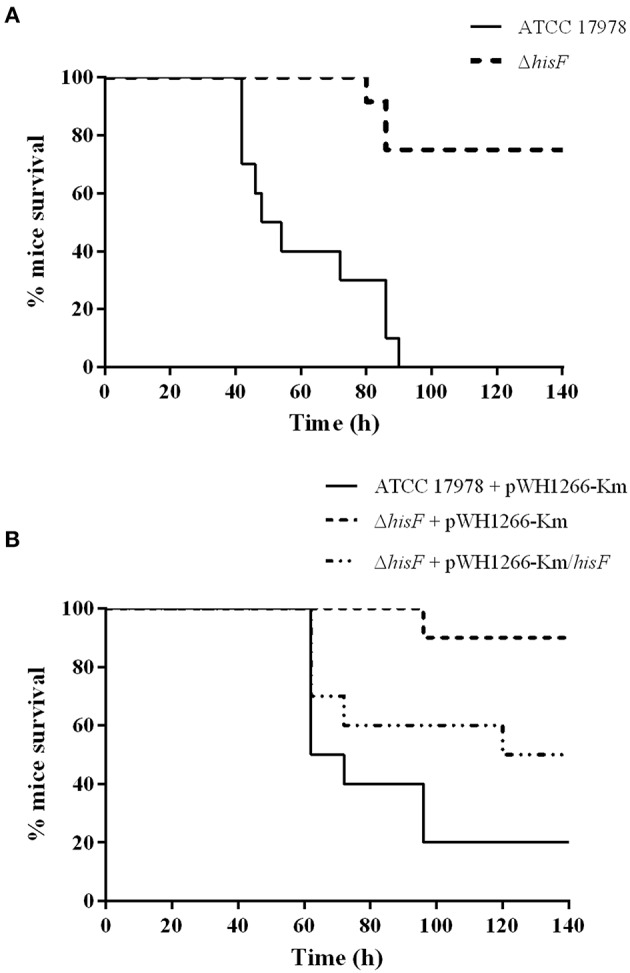
Survival rate of mice in a pneumonia model (*N* = 10). **(A)** The parental ATCC 17978 and the mutant Δ*hisF A. baumannii* strains. Survival was significantly higher in mice infected with the Δ*hisF* mutant (*p* < 0.01). **(B)** The ATCC 17978 + pWH1266-Km (empty plasmid), the Δ*hisF* + pWH1266-Km, and the complemented Δ*hisF A. baumannii* strains. Survival was significantly lower in mice infected with the complemented than in the Δ*hisF* strain harboring the empty plasmid (*p* < 0.05). There were no significant differences in survival between mice infected with ATCC 17978 + pWH1266-km and Δ*hisF* complemented strains.

Remarkably, the murine pneumonia model showed that the survival rate of mice infected with the Δ*hisF* mutant strain was significantly higher than that of mice infected with the parental strain (*p* < 0.001, Figure 1A). The lack of the *hisF* gene led to an important loss of virulence in the *A. baumannii* ATCC 17978 strain. Conversely, no such differences were observed in the murine sepsis or in the *G. mellonella* infection models (Figure S3). When bacterial burden in lungs was analyzed at 20 h, results confirmed the relevant decrease of virulence in the experimental murine pneumonia. The lungs of mice infected with the Δ*hisF* mutant showed two logarithms less bacterial burden than those infected with the parental strain (*p* < 0.01, Figure S4).

Then, complementation of the mutant strain with the A1S_3245 gene (Δ*hisF* complemented strain) was performed by expressing the *hisF* gene from the pWH1266-Km vector. In a parallel assay, virulence of both the mutant and the parental strains harboring the empty plasmid and the mutant containing the plasmid harboring the *hisF* gene were analyzed using the murine pneumonia model. No significant differences were observed between the *A. baumannii* wild type strain harboring the empty pWH1266-Km vector and the Δ*hisF* mutant strain complemented. However, the virulence of the Δ*hisF* mutant strain complemented was higher than that of the Δ*hisF* mutant strain harboring the empty plasmid (*p* < 0.05, Figure 1B). Overall the data indicated that the *hisF* gene expressed from the plasmid partly restored the wild type phenotype. It should be noted that carrying the pWH1266-Km plasmid implies an important metabolic load to the bacterium, which affects its growth rate and its fitness (Álvarez-Fraga et al., 2018). As shown in Figure S2C, the strains carrying the pWH1266-Km plasmid decreased their fitness, which, in turn, affects their virulence. Therefore, the survival rate of mice infected with strains harboring the pWH1266-Km plasmid was higher than that of mice infected with strains without plasmids, for the same bacterial inoculum. However, as shown in Figure S2C no fitness differences were observed between the ATCC 17978 and the Δ*hisF* strains or between the ATCC 17978 carrying pWH1266-Km, the Δ*hisF* mutant strain carrying pWH1266-Km and the Δ*hisF* mutant strain complemented. Thus, the decreased virulence of Δ*hisF* mutants in murine pneumonia assays is exclusively attributable to the inactivation of *hisF* gene and not to the presence/absence of the pWH1266-Km plasmid.

The transformation of PRFAR by HisF produces AICAR, used in *de novo* purine biosynthesis and ImGP, used in histidine biosynthesis (Figure S1). Purine nucleotides can be synthesized trough two distinct pathways. First, purines can be synthesized *de novo*, attaching a formyl group to AICAR to produce IMP (inosine monophosphate) and later AMP and GMP (guanosine monophosphate). Alternatively, purine bases can be salvaged and recycled by the hydrolytic degradation of nucleic acids and nucleotides. However, to synthetize histidine the HisF protein and ImGP are essential. Thus, histidine auxotrophy assays were performed to demonstrate the implication of A1S_3245 in AICAR and ImGP production. As reflected in Figure S5, while the ATCC 17978 strain was able to grow in M9 minimal medium, the Δ*hisF* mutant strain was unable to grow, decreasing the number of viable bacteria at 24 h. The addition of 0.5 mM of histidine to the M9 medium does not affected the growth rate of the parental strain but increased the growth rate of the Δ*hisF* mutant. The mutant showed a growth rate very similar to the parental strain, thus displaying the later auxotrophy by histidine.

Lungs are particularly susceptible to infection because of the huge epithelial surface in contact with inspired air. Thus, the respiratory tract must possess defense mechanisms such as the anatomical barriers of the nose or the phagocytes in alveoli. The cytokine IL-6 is involved in regulating inflammatory responses during bacterial infection, and high IL-6 concentrations are detected in BAL fluids from patients with pneumonia (Dehoux et al., 1994). In murine models of pneumonia, IL-6 has been described as being involved in antibacterial host defense and in regulating the cytokine network in lungs (van der Poll et al., 1997). Thus, the acute pulmonary inflammatory response caused by local exposure to bacterial lipopolysaccharide is regulated by inflammatory mediators such as IL-6.

**Figure 2:**
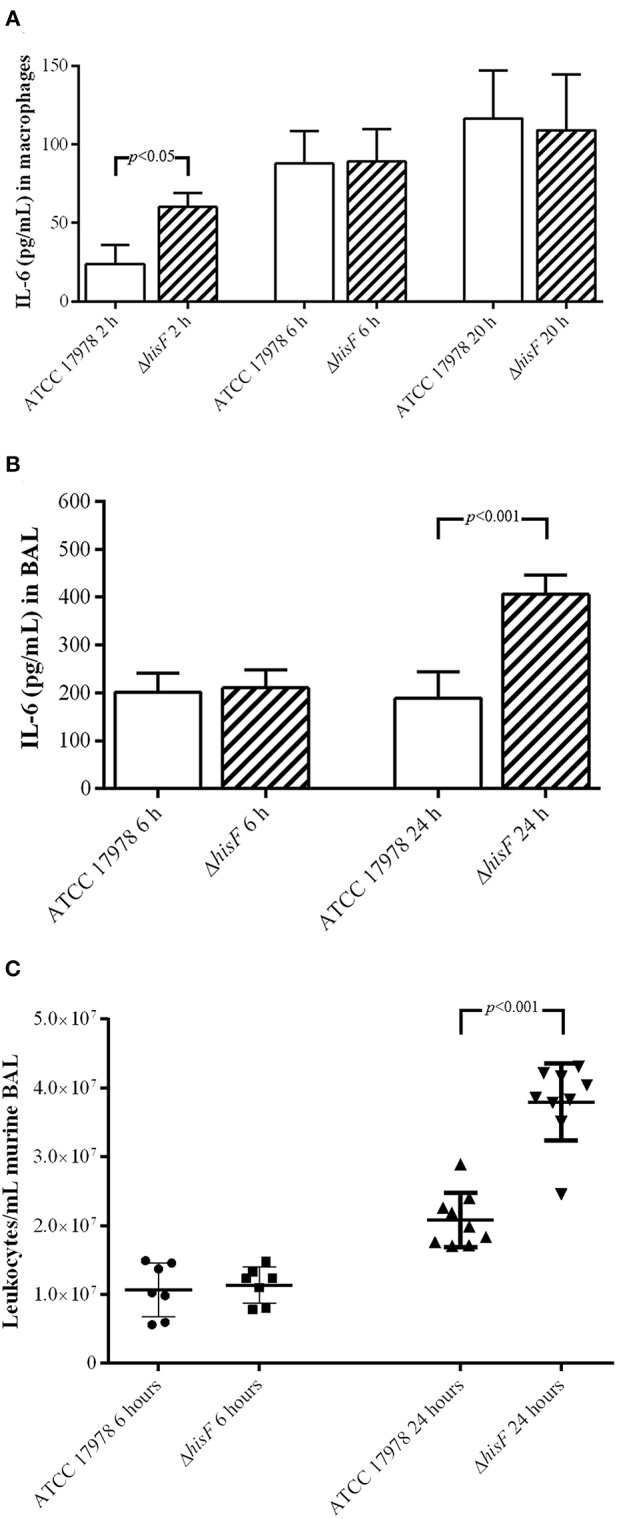
**(A)** Amount of IL-6 at 2, 6, and 20 h post-infection in the cell-free supernatant of macrophages RAW 264.7 (*N* = 5) infected with the parental ATCC 17978 and the Δ*hisF* mutant *A. baumannii* strains. **(B)** Amount of IL-6 at 6 and 24 h post-infection in BAL fluid from mice lungs (*N* = 7) infected with the parental ATCC 17978 and the Δ*hisF* mutant *A. baumannii* strains. **(C)** Total leukocyte counts in BAL fluid from mice lungs infected with the parental ATCC 17978 and the Δ*hisF* mutant *A. baumannii* strains, at 6 h (*N* = 7) and 24 h (*N* = 9) post-infection.

In this study, immunoassays performed to detect the cytokine IL-6 in macrophages RAW 264.7 infected with the parental and the mutant strains, indicated that the macrophages differentially secreted IL-6 at 2 h (*p* < 0.01). In this model of *in vitro* infection the mutant strain was able to induce faster the IL-6 secretion, starting the pro-inflammatory response before than the parental strain. However, at 6 and 20 h, the IL-6 levels secreted by macrophages RAW 264.7 were similar in infections caused by both strains (Figure 2A). Thus, the increase of AICAR biosynthesis is involved in a delayed IL-6 secretion. ELISA analysis of BAL fluids from the murine pneumonia model revealed that the IL-6 concentration was higher in BAL fluids from mice infected with the Δ*hisF* mutant strain than in those infected with the parental strain (*p* < 0.001) at 24 h post-infection, whereas, no significant difference was were observed in murine BAL fluids obtained at an earlier stage post-infection (6 h) (Figure 2B).

IL-6 is one of the most discriminative markers for definition and evaluation of recovery in patients with pneumonia, being the serum levels of IL-6 in mild or severe pneumonia infections higher (de Brito et al., 2016). During the initial phases of pneumonia, alveolar macrophages produce a variety of pro-inflammatory cytokines such as IL-6, whose role is to both attract and activate polymorphonuclear leukocytes, necessary for local bacterial defense and clearance (Bordon et al., 2013). Thus, noticing an increase of IL-6 secretion in the infection of the Δ*hisF* mutant, we performed a study of leukocytes recruitment in lung infection caused by the parental ATCC 17978 and the Δ*hisF* mutant strains. Leukocyte counts in BAL fluids obtained from the lungs of mice infected with the Δ*hisF* mutant were almost two times higher than in those infected with the parental strain (*p* < 0.001) at 24 h (Figure 2C). Microscopic visualization also allowed identifying the different types of leukocytes. However, although differences were found in the total number of leukocytes in the infections, no differences were observed between each cell type in infections caused by the parental (neutrophils 69%, lymphocytes 16%, macrophages 8%, eosinophils 6%, or basophils 1%) and the mutant strain (neutrophils 61%, lymphocytes 26%, macrophages 3%, eosinophils 7%, or basophils 3%). In contrast to the infection in macrophages, which showed differences at the very early stage post-infection, in the pneumonia model the significant differences were observed at 24 h post-infection, both in IL-6 production and in leukocyte cells counts. Thus, expression and recruitment of cytokines take longer in the *in vivo* model of infection than in the *in vitro* model of cultured macrophages.

**Figure 3:**
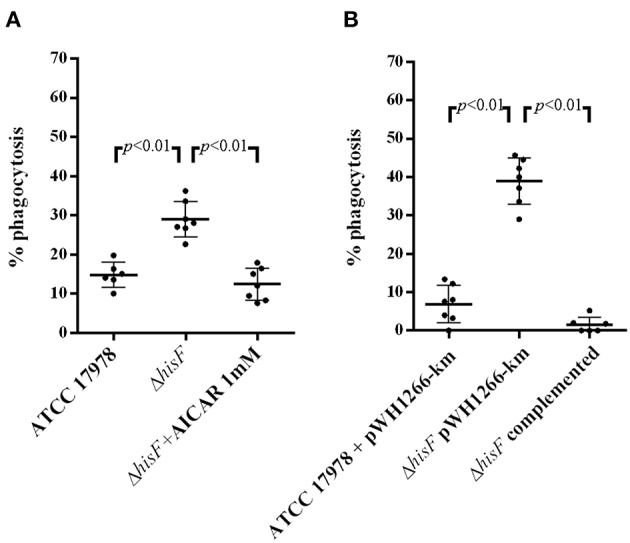
*In vitro* phagocytosis assays with macrophages RAW 264.7. **(A)** Per cent killing of *A. baumannii* ATCC 17978, the Δ*hisF* mutant, and the Δ*hisF* mutant in presence of AICAR 1 mM (*N* = 6). **(B)** Per cent killing of *A. baumannii* ATCC 17978 + pWH1266-Km (empty plasmid), the Δ*hisF* + pWH1266-Km, and the Δ*hisF* complemented strains (*N* = 6).

In order to correlate with protection, IL-6 measured by ELISA was compared with the implication of *hisF* and AICAR in phagocytic activity assays. As illustrated in Figure 3A, there was higher phagocytosis of the Δ*hisF* mutant strain (28% phagocytosis) than of the parental strain (14%, *p* < 0.01). The protective effect of HisF was confirmed in assays with the *A. baumannii* strains carrying the plasmid pWH1266-Km. Thus, the Δ*hisF* + pWH1266-Km mutant showed more susceptibility to macrophages (39% phagocytosis) that the ATCC 17978 + pWH1266-Km (6%, *p* > 0.01), concordantly with the previous result. Relevantly the complementation with the *hisF* gene increased the resistance to the phagocytosis, the Δ*hisF* complemented strain showed very similar number of viable bacteria (2% phagocytosis) to the ATCC 17978 + pWH1266-Km strain (Figure 3B). Finally, significant phagocytosis protection was observed when AICAR at 1 mM was used in the assays with the Δ*hisF* + pWH1266-km mutant showing phagocytosis percentages similar to those of the parental ATCC 17978 in absence of AICAR (Figure 3A). Thus, it is clear than both the genetic complementation with the *hisF* gene or the AICAR addition to the culture medium, increase the resistance to macrophages of the *A. baumannii* strain with the inactivated *hisF* gene.

The results of infection of RAW 264.7 macrophages and of *in vivo* pneumonia experiments using the parental and the isogenic mutant strains suggested that HisF participates in the early and acute inflammatory responses and host defense against *A. baumannii* infection, inhibiting initiation of the innate immune cell recruitment in lungs. Moreover, the data obtained in this study suggest that conversion of PRFAR by the heterodimeric protein HisHF, in ImGP and AICAR, plays a key role in lung infection caused by *A. baumannii*. AICAR expression, which is involved in AMPK phosphorylation, can be used by the bacteria to reduce the host immune response and favor infection. The role of AICAR and AMPK in inflammation has previously been described (Jhun et al., 2004; Peairs et al., 2009), although AICAR is known to exert various other effects such as regulation of cell proliferation and apoptosis, *via* both AMPK-dependent and independent mechanisms (Campàs et al., 2003; López et al., 2003). The adenosine kinase inhibitor 5′-iodotubercidin has been used to prevent the AICAR-mediated inhibition of inflammation cascade signaling, suggesting that AMPK activation may be considered a potential therapeutic target in inflammatory diseases (Peairs et al., 2009).

Significant differences between the wild-type and the Δ*hisF* mutant strains were observed in relation to survival in the murine pneumonia model, but not in the sepsis model. Inactivation of the *hisF* gene led to disappearance of *A. baumannii*-associated virulence, which did not happen in the bacteraemia model. This can be attributed to the differences between the local and the systemic inflammation due to the immune response. The importance of pro-inflammatory cytokines in host defense during pneumonia, which have different roles in local inflammation than in the systemic compartment, has been well-described (Schultz and Poll, 2005; Madigan et al., 2014). Neutrophils are the first inflammatory cells to arrive at the infection site, attracted by chemoattractants such as interleukins. Neutrophils ingest the damaged cells and also attract macrophages, which carry out phagocytosis. The macrophages release inflammation-inducing cytokines, such as IL-1 or IL-6, which increase vascular permeability, swelling, and local heat. As a result, the pathogen is rapidly located and destroyed by the recruited macrophages and neutrophils. However, in bacteraemia models, the infection is not localized, leading to extended systemic inflammation. Septic shock occurs when the inflammatory response disseminates inflammatory cells and mediators through the circulatory and lymphatic systems. Thus, although local production of pro-inflammatory cytokines contributes greatly to host defense during local infections in the lung, the excessive production of pro-inflammatory cytokines at the systemic level causes organ failure and death in animal models (Schultz and Poll, 2005; Madigan et al., 2014). We suggest that the different virulence phenotypes found in both infection models are at least partly due to the different consequences of the pro-inflammatory interleukin-mediated inflammatory processes.

To the best of our knowledge, this is the first study describing the interplay between HisF and the innate immune response in lungs during bacterial pathogenesis. Further studies and detailed analysis of the inflammatory response are needed for a better understanding of the role of HisF in the *A. baumannii* pathogenesis and host defense.

In conclusion, the study findings demonstrated that the lack of HisF in the pneumonia infection of immunocompetent BALB/c mice caused by ATCC 17978 *A. baumannii* induces a sub-lethal infection. Complementation with the original *hisF* gene in the Δ*hisF* mutant increased its virulence in the experimental pneumonia model. HisF is involved in inhibition of the recruitment of innate immune cells, as well as in the production of the proinflammatory cytokine IL-6. Thus, the *hisF* gene from *A. baumannii* ATCC 17978, which is over-expressed during the course of a pneumonia infection, is essential for full virulence of the strain in lung infection. The *hisF* gene is therefore an alternative and useful tool for future pathogenesis studies of *A. baumannii-*associated pneumonia and for identifying and characterizing important virulence factors, and it thus represents a potential target for evaluating new antimicrobial therapies. In light of the study findings, expression of the *hisF* gene seems to decrease the innate immunity and the inflammatory responses, which may partly explain the ability of the pathogen to persist in the lung.

## Data Availability

Publicly available datasets were analyzed in this study. This data can be found here: https://www.ncbi.nlm.nih.gov/geo/query/acc.cgi?acc=GSE100552.

## Author Contributions

MM-G, KC-P, and CL-M performed phenotypic experiments and inmunoassays. JV-U and JV performed animal models. LÁ-F performed transcriptomics and mutant construction. GB supervised the experiments. AB and MP designed and supervised the experiments and wrote the manuscript. All authors discussed the results and contributed to the final manuscript.

### Conflict of Interest Statement

The authors declare that the research was conducted in the absence of any commercial or financial relationships that could be construed as a potential conflict of interest.
