# Supplementary material for: Involvement of HisF in the Persistence of Acinetobacter baumannii During a Pneumonia Infection
Source: Front Cell Infect Microbiol. 2019 Aug 29;9:310. doi: 10.3389/fcimb.2019.00310 (PMC6727670; doi:10.3389/fcimb.2019.00310)

Supplementary material

Table S1. Oligonucleotides used in the present work.

**Primer name**	**Sequence**	**Use in the study**
A1S_3245UPFw	ttgcggccgcaagtcacggaacaaatctgag	Construction of the Δ*hisF* mutant
A1S_3245UPRv	ccggaattccgggttaatactcactttgtctg	Construction of the Δ*hisF* mutant
A1S_3245DOWN	ccggaattccggtatcgaaatgcgcttgtaag	Construction of the Δ*hisF* mutant
A1S_3245DOWNRv	cgcggatccgcggtttggcaatttctagccc	Construction of the Δ*hisF* mutant
A1S_3245intUP	gcctgtgtaggaactttca	Confirmation of the deletion of the *hisF* gene
A1S_3245intDOWN	ccactctctcaagctgta	Confirmation of the deletion of the *hisF* gene
A1S_3245FwEcoRVCompl	cccgatatcatgcttgctaaacgtattat	Cloning the *hisF* gene into the pWH1266-Km plasmid to complement the Δ*hisF* strain
A1S_3245RvBamHICompl	cccggatccttacaagcgcatttcgatac	Cloning the *hisF* gene into the pWH1266-Km plasmid to complement the Δ*hisF* strain

Table S2. Antibiotic susceptibility profile of *A. baumannii* ATCC 17978 and the *ΔhisF* mutant.

	**Zone diameter mean difference (mm)**	
**Antibiotic**	**ATCC 17978**	***ΔhisF***
Ampicillin	9	9
Cefoxitin	9	9
Ceftazidime	20	21
Cefepime	22	23
Imipenem	29	31
Tigecycline	22	24
Rifampicin	11	11
Colistin	13	14
Ciprofloxacin	25	27
Gentamicin	24	26

Figure S1. Reactions catalyzed by HisH and HisF. The ammonia molecule required for this reaction is provided by the glutaminase HisH which transfers nitrogen from glutamine to form glutamate. Later, PRFAR is converted by HisF into ImGP and AICAR. The second product of the reaction, AICAR, is further used in *de novo* purine biosynthesis and AMPK activation.

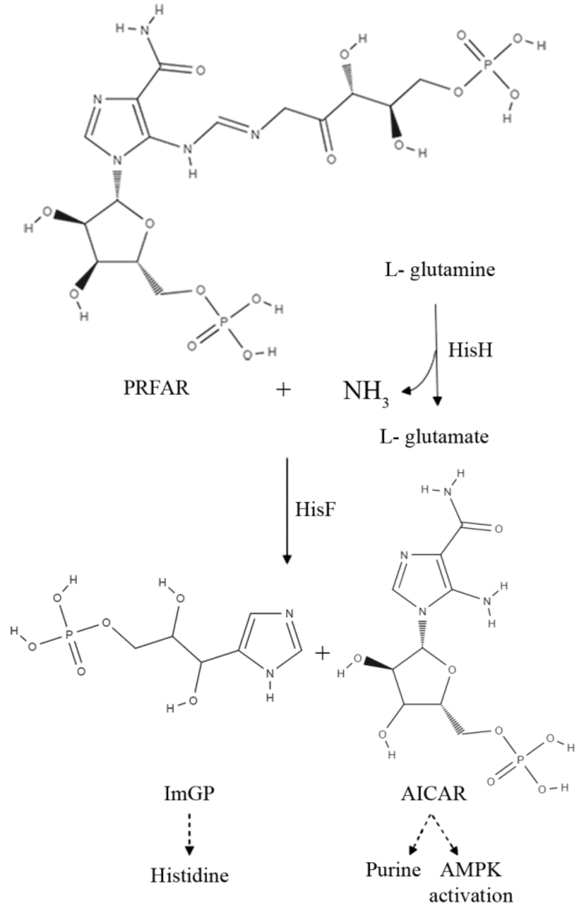

Figure S2. *In vitro* assays. A) Quantification of the biofilm formation ability of *A. baumannii* ATCC 17978 strain and the Δ*hisF* mutant derivative strain (N=6, bars represent the standard deviations). B) Quantification of the bacterial adhesion ability to A549 cells of *A. baumannii* ATCC 17978 strain and the mutant derivative strain Δ*hisF* (N=6, bars represent the standard deviations). C) Growth curves of the ATCC 17978 strain, the isogenic mutant derivative strain Δ*hisF*, ATCC 17978 + pWH1266-Km, Δ*hisF* + pWH1266-Km and Δ*hisF* complemented strains. Data correspond to the mean of 4 replicates and bars represent the standard deviations. D) Fluorescence microscopy images of human alveolar A549 cells infected by ATCC 17978 and Δ*hisF* strains after 16 h post-infection.

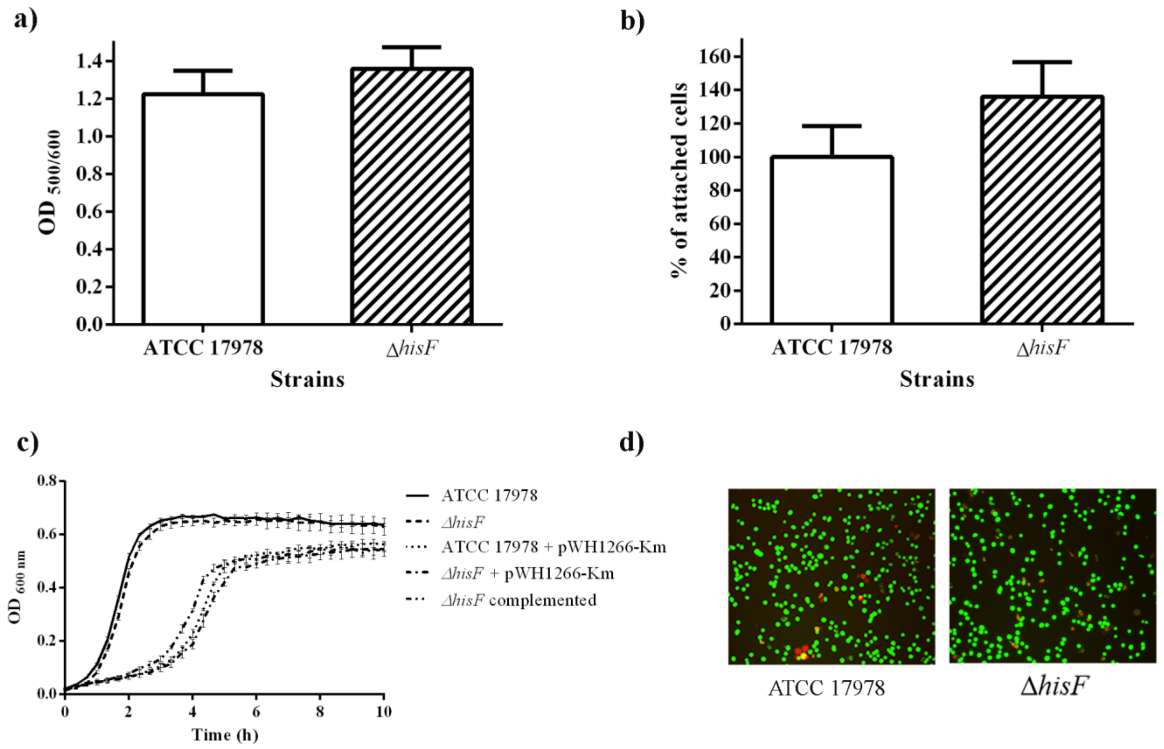

Figure S3. *In vivo* assays. A) Experimental murine model of bacteraemia performed with the *A. baumannii* ATCC 17978 and the Δ*hisF* isogenic mutant strains (N=10). B) *G. mellonella* larvae infection model using the *A. baumannii* ATCC 17978 and the Δ*hisF* isogenic mutant strains. Non-infected larvae (PBS) were used as control (N=10).

a)

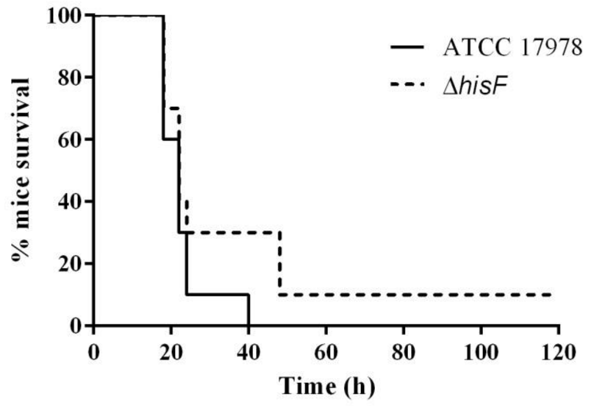

b)

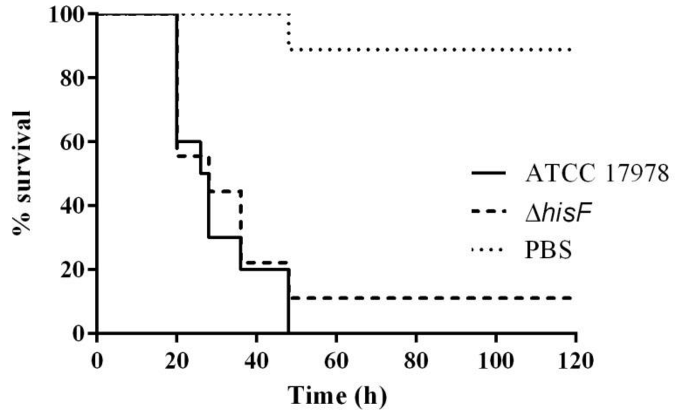

Figure S4. Bacterial burden in lungs. Bacterial load determination in lungs of mice infected with the ATCC 17978 and the Δ*hisF* mutant strains (N=8) (p<0.01).

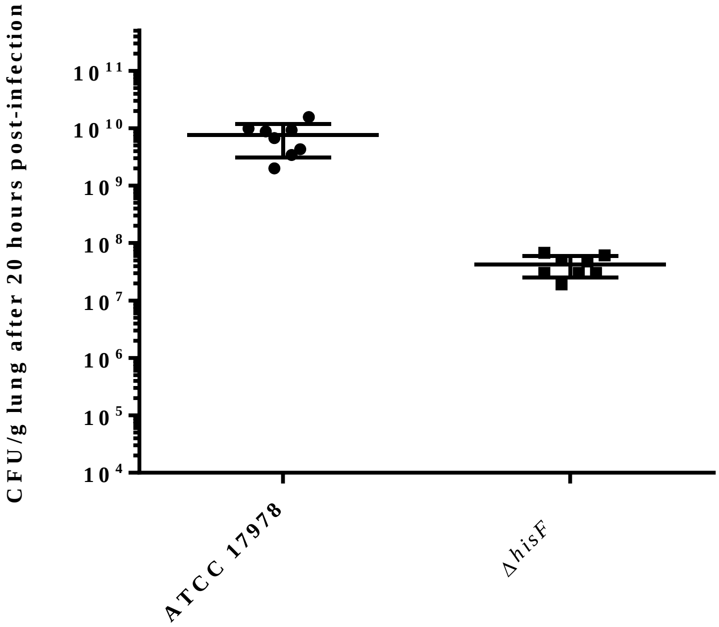

Figure S5. Determination of histidine auxotrophy. Curves of viable bacteria were performed with the ATCC 17978 and the Δ*hisF* mutant strains in M9 minimal medium in presence and absence of histidine.

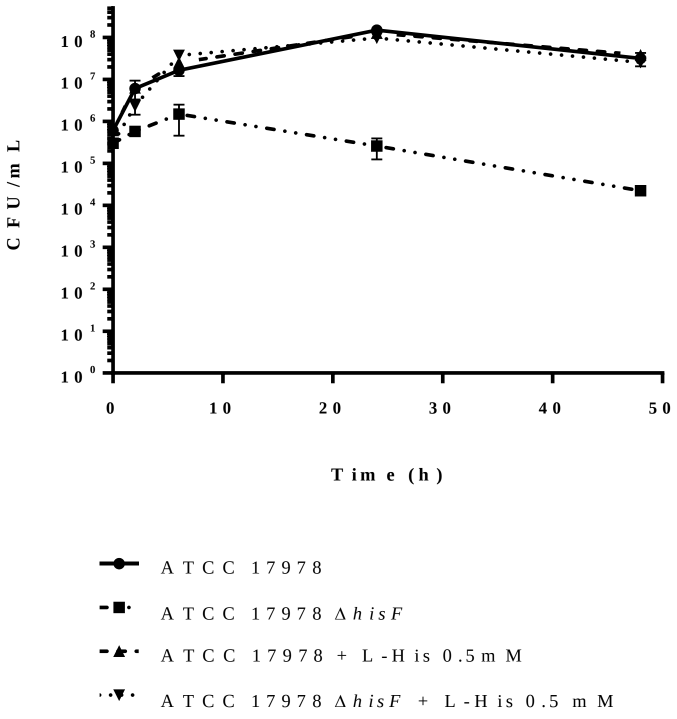

Supplement: Supplementary file 1 [file Presentation_1.pdf]
